# Exposure to maternal high-fat diet induces extensive changes in the brain of adult offspring

**DOI:** 10.1038/s41398-021-01274-1

**Published:** 2021-03-02

**Authors:** Darren J. Fernandes, Shoshana Spring, Anna R. Roy, Lily R. Qiu, Yohan Yee, Brian J. Nieman, Jason P. Lerch, Mark R. Palmert

**Affiliations:** 1 Mouse Imaging Centre, The Hospital for Sick Children, Toronto, ON Canada; 2 Department of Medical Biophysics, University of Toronto, Toronto, ON Canada; 3 Neurosciences and Mental Health, The Hospital for Sick Children, Toronto, ON Canada; 4 Division of Endocrinology, The Hospital for Sick Children, Toronto, ON Canada; 5 Wellcome Centre for Integrative Neuroimaging, University of Oxford, Oxford, UK; 6 Translational Medicine, Hospital for Sick Children, Toronto, ON Canada; 7 Ontario Institute for Cancer Research, Toronto, ON Canada; 8 Department of Paediatrics and Physiology, University of Toronto, Toronto, ON Canada

**Keywords:** Neuroscience, Genetics, Physiology

## Abstract

Maternal environmental exposures, such as high-fat diets, diabetes and obesity, can induce long-term effects in offspring. These effects include increased risk of neurodevelopmental disorders (NDDs) including autism spectrum disorder (ASD), depression and anxiety. The mechanisms underlying these late-life neurologic effects are unknown. In this article, we measured changes in the offspring brain and determined which brain regions are sensitive to maternal metabolic milieu and therefore may mediate NDD risk. We showed that mice exposed to a maternal high-fat diet display extensive brain changes in adulthood despite being switched to a low-fat diet at weaning. Brain regions impacted by early-life diet include the extended amygdalar system, which plays an important role in reward-seeking behaviour. Genes preferentially expressed in these regions have functions related to feeding behaviour, while also being implicated in human NDDs, such as autism. Our data demonstrated that exposure to maternal high-fat diet in early-life leads to brain alterations that persist into adulthood, even after dietary modifications.

## Introduction

The prevalence of obesity is increasing among women of reproductive age^1,2^. Overweight or obese mothers may predispose their children to adverse health outcomes – such as diabetes, obesity and coronary heart disease^3–5^. More recently, the maternal environment has been recognised as an important factor in offspring brain development^6–8^. Evidence has shown that the perinatal exposure to maternal obesity and associated metabolic abnormalities increases the risk of neurodevelopmental disorders (NDDs) in offspring, including attention deficit hyperactivity disorder, autism spectrum disorders, anxiety, depression, schizophrenia and eating disorders^9–12^.

Due to the slow emergence of NDDs in humans, it is difficult to study mechanisms driving the association between maternal environment and NDDs in offspring. Mouse models provide an important opportunity to explore this link; the effects of typical human “Western” diets (which consists of 30–40% of calories from fats), including characteristic metabolic abnormalities, have been successfully recapitulated in mice fed diets consisting of 40–60% fat^13–15^. Furthermore, offspring of rodents fed high-fat diet exhibit a wide range of behavioural changes consistent with NDDs^16,17^. For example, rodents exposed to high-fat diet before weaning displayed increased susceptibility to depression-like behaviours^18^, increased anxiety^19^ and impaired spatial learning^20^.

While the relationship between maternal diet and offspring NDD risk is likely to be mediated by developmental changes in particular brain structures, a systematic measure of alterations across the whole brain has not previously been reported. Since mice and humans have many homologous brain structures, the brain regions affected by maternal diet in mice may identify relevant structures in humans. Viral labelling of hippocampal neurons has already shown that dendritic arborization is impaired in offspring of mice fed high-fat diet^20^. However, the extent to which the rest of the brain may also be altered is unclear. Magnetic resonance (MR) imaging allows for the study of the whole brain in a high-throughput manner with sufficient resolution to quantify regional changes in volume or morphology^21^.

Our aim was to explore the effects of maternal diet on offspring brain structure. Adult mice were fed one of three diets during breeding, gestation and lactation: a low-fat diet with 10% of calories from fat (LF10) or high-fat diets with 45% or 60% of calories from fat (HF45 and HF60, respectively). After weaning, all offspring were raised on the low-fat diet until postnatal day 65 (P65), corresponding to early adulthood. MR imaging was used to measure neuroanatomical changes in adult offspring attributable to pre-weaning high-fat diet exposure. The Allen Brain Institute’s gene expression atlas was then used to identify which genes had spatial expression patterns consistent with the affected neuroanatomy.

## Methods

### Maternal and offspring diet

Five-week-old C57Bl6/J mice, raised on a 18% protein diet (diet 2918, Envigo, Indianapolis, IN), were acclimated to one of three new diets (Research Diets Inc., New Brunswick, NJ) for 6 weeks:

1. D12450Bi: 10% kcal from fat (LF10)
2. D12451i: 45% kcal from fat (HF45)
3. D12492i: 60% kcal from fat (HF60)

Block randomisation was used for allocation of cages (each containing 3–5 mice) to experimental groups. Investigators were not blinded when assessing experimental outcomes. After diet acclimation, diet-fed-matched females and males were paired for mating. Impregnated females were individually housed and remained on their respective diets throughout breeding, gestation and lactation. Their offspring pups were weaned at P21 and group-housed with 4–5 animals per cage. All offspring mice were fed the LF10 diet ad libitum after weaning and throughout adulthood. All animal experiments were approved by The Centre for Phenogenomics (TCP) Animal Care Committee (AUP 17-0175H) in accordance with recommendations of the Canadian Council on Animal Care (CCAC), the requirements under the Animals for Research Act, RSO 1980, and the TCP Committee Policies and Guidelines.

### Data analysis

R was used to conduct statistical analyses^22,23^. Linear mixed-effects (LME) models were fitted using maximum-likelihood^24,25^. Significance of predictor and group differences were assessed using Wilk’s theorem^26^ and tukey-adjusted *p*-values^27^, respectively. 95%-confidence intervals were estimated from 1000-sample parametric-bootstrap. Center values in all plots represent model predictions with error bars and shaded regions representing 95%-confidence intervals.

### Growth rates, body composition and metabolic testing

Dam weights were recorded weekly throughout the 6-week diet acclimation period: *n* = 18,15,18 (LF10,HF45,HF60). After weaning, offspring mice were weighed regularly until P65. The number of female (F) and male (M) offspring from dams (D) in each group were: *n* = 37F/36M/8D, 24F/37M/8D, 15F/17M/6D (LF10, HF45, HF60). Maternal data was fitted with LME models: fixed effects (FE) of diet (categorical), time-on-diet (cubic spline) and all interactions; random effect (RE) of mouse-specific intercept and time-on-diet. Offspring data was fitted with a similar LME models, but had an additional FE of sex (all interactions) and RE of time-on-diet (quartic natural spline).

A bench-top NMR analyzer (Minispec, Bruker, Bellerica, MA) was used to distinguish fat tissue from lean tissue in vivo^28^. Dams were assessed for body composition after diet acclimation at ~11 weeks-of-age: *n* = 18, 15, 18 (LF10, HF45, HF60). Offspring mice were assessed for body composition at ~9 weeks-of-age: *n* = 11 F/11 M, 11 F/11 M, 12 F/13 M (LF10, HF45, HF60). ANOVA was conducted on the data with predictors of diet, sex (for offspring data only) and all interactions; and Tukey test used to identify significant group differences.

To conduct glucose tolerance testing (GTT)^28,29^, mice were housed without food for 6 h, blood glucose levels were checked, 10% glucose solution was administered via intraperitoneal (IP) injection (dose 0.01 mL/g) and glucose levels were checked again at 30, 60 and 120 min post-injection. GTT was conducted on dams after diet acclimation (*n* = 12/group) and adult offspring at 9 weeks-of-age: *n* = 8F/8M, 8F/8M, 6F/8M (LF10, HF45, HF60). Data was fitted using LME models: FE of post-injection time (categorical variable), diet, sex (for offspring only) and all interactions; RE of mouse-specific intercept.

### Brain perfusion, fixation and image acquisition

Fixed brain samples were prepared for MR imaging^30^ to assess neuroanatomy of dams (*n* = 8/group, two mice in the HF60 group did not give live births but were included) and offspring: *n* = 17F/17M, 17F/17M, 17F/16M (LF10, HF45, HF60). All adult offspring from the HF60 group were chosen and a random portion of the litters in the other diet groups were chosen to balance the numbers in each group. A minimum of *n* = 8/group was chosen as this allows for detection of 3% volumetric differences between groups at a critical significance and power level of 5% and 80%, respectively^31^. By comparison, the basal forebrain was ~5% larger in pups fed the HF60 diet. P127 ± 9 dams and P65 ± 3 offspring (median ± range) were anaesthetised, transcardially perfused and decapitated. Brains (kept within the skulls) were placed in a solution of paraformaldehyde (PFA) and Prohance® (gadoteridol, Bracco Diagnostics Inc., Princeton, NJ) for fixation. A multichannel 7.0-T scanner with a 40-cm diameter bore magnet (Varian Inc., Palo Alto, CA) was used to acquire MR images at 40-µm-isotropic resolution^32^ for 16 brain samples concurrently^21^. Scan parameters: T2W 3D FSE cylindrical *k*-space acquisition sequence, TR/TE/ETL = 350 ms/12 ms/6, two averages, FOV/matrix-size = 20 × 20 × 25 mm/504 × 504 × 630, total-imaging-time = 14 h^32^.

### Image registration and analysis

Using an image registration pipeline^31,33^, all 24 dam brains and 101 offspring brains were registered together using the mni_autoreg^34^, ANTs^35^ and pydpiper toolkits^36^. The pipeline’s outputs included the consensus average, and jacobian determinants quantifying the volumetric difference between each MR image and the average. The MAGeT pipeline^37^ was used to segment images using a published atlas^38–42^.

To assess the effect of diet on neuroanatomy, an ANOVA was conducted on the log of the jacobian determinants at every voxel: predictors of diet, sex (of offspring only) and their interaction. To assess significance of group differences for particular structures, a similar ANOVA was conducted on the structure volumes and tukey-adjusted *p*-values were computed. To estimate fat-dosage effects, a linear model was fitted to the log jacobian determinants at every voxel: predictors of dietary fat-percentage (continuous variable), sex and their interaction. All statistics were corrected for multiple comparisons using false-discovery-rate (FDR)^43^. Normality and homoscedasticity of data were assessed for each structure using the Kolmogorov-Smirnov and Breusch-Pagan test, respectively, and found to be valid (minimum FDR of 0.25 and 0.16, respectively).

### Spatial gene expression analysis

Spatial gene expression analysis was conducted similarly to previously published studies using the ABIgeneRMINC package^44^. Briefly, our MR average was aligned to the Allen Brain Institute (ABI) Gene Expression Atlas using ANTs^35^. The top 65% of voxels with the highest diet F-statistics (which corresponds to 1% FDR) constituted the region-of-interest (ROI). ROI with top 60–70% voxels showed similar results. Preferential gene expression was quantified by a fold-change measure – gene expression signal in the ROI divided by gene expression in the whole brain – and was computed for all genes, except those with data spanning under 20% of the brain. Preferential expression of developmental gene expression data was *Z*-transformed and was clustered using *k*-means. To avoid clustering genes with ubiquitous expression, only genes that had at least 5% fold-change at any time point were clustered.

Gene Ontology (GO) enrichment analysis was conducted on the top 4000 genes with the greatest fold-change in adulthood using the GOrilla web application^45^ (background set including all genes). Similar results were seen for the top 3000–5000 genes and ranked list of all genes. Significance of GO terms were assessed using permutation testing (10,000 iterations), where, in each permutation, the association between subjects and diets were randomised. Disease processes associated with gene preferential expression were determined using the Mouse Genome Informatics (MGI) database^46,47^. Human disease terms containing fewer than 15 homologous mouse genes were excluded from the analysis (leaving 13 disease terms in all). For each disease, the fold-change of all the disease-associated mouse genes were averaged and this was the test statistic. Significance was assessed using permutation testing (10,000 iterations), where, in each permutation, the association between diseases and genes were randomised. *P*-values were adjusted using Bonferroni correction^48^. To validate findings related to autism genes, the Simons Foundation Autism Research Initiative (SFARI) database was used to find genes related to autism mouse models. Gene data was downloaded on April 7,2020.

## Results

### Dams fed high-fat diet showed significant changes in physiology but not brain structure

**Fig. 1:**
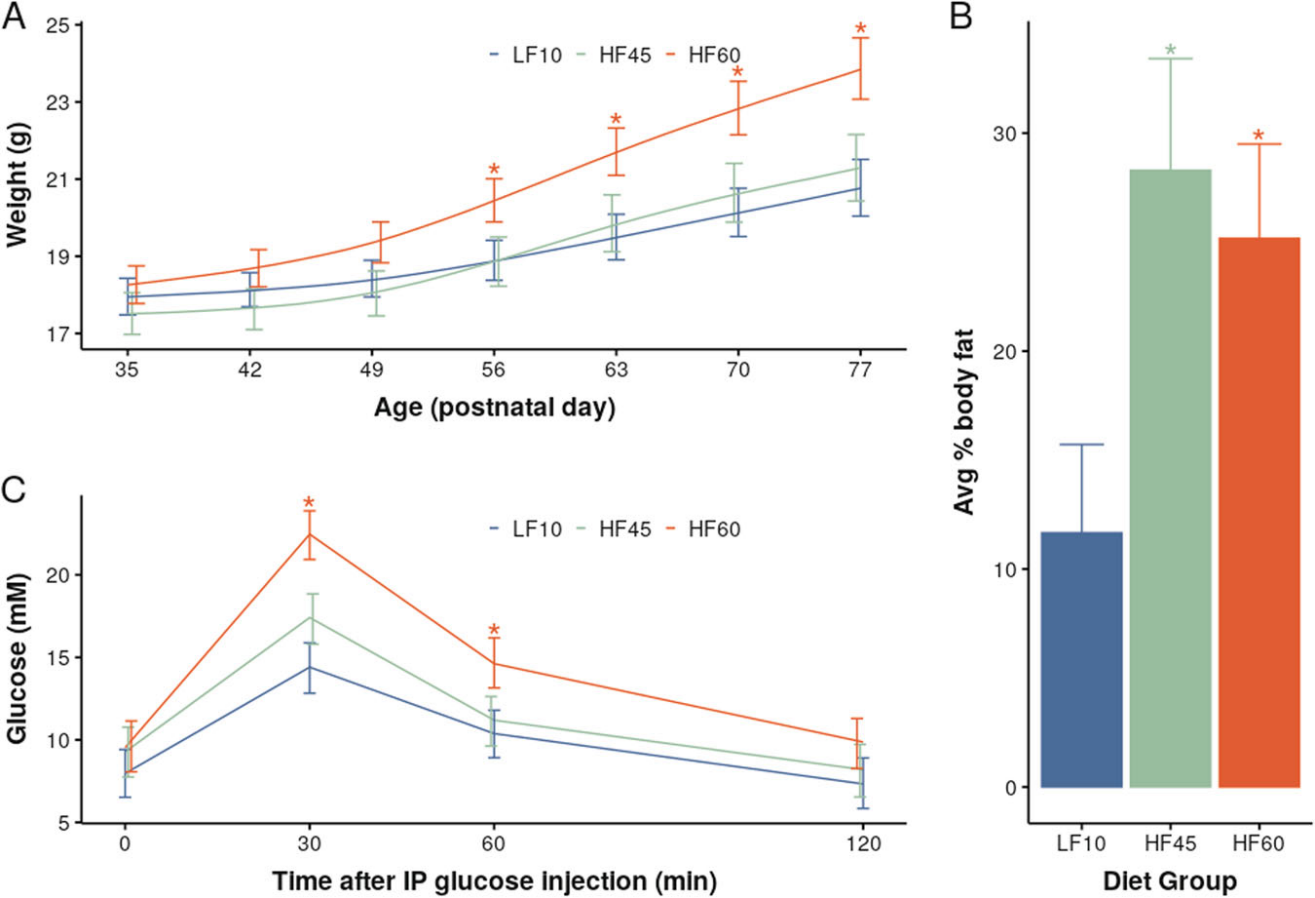
High-fat diet induced metabolic changes in dams. **A** Body weights were significantly affected by diet (*χ*^2^_8_ = 41.8, *p* < 10^−5^), primarily driven by a diet-time interaction (*χ*^2^_6_ = 36.9, *p* < 10^−5^). A significant difference between LF10 and HF60 groups emerged at ~2–3 weeks after the experimental diet was introduced. **B** Average body-fat percentage was significantly affected by diet (HF45-LF10 difference = 16.6%, *p* < 10^−3^; HF60-LF10 difference = 13.5%, *p* < 10^−3^). **C** GTT showed a significant difference in glucose levels 30 min. (difference = 8.04 mM, *p* < 10^−8^) and 60 min. (difference= 4.25 mM, *p* < 10^−2^) between the LF10 and the HF60 groups. All bars represent 95% confidence intervals. Asterisks represent a significant difference from LF10 (*p* < 0.05).

To verify effectiveness of the dietary manipulation, dams’ body weight, body composition, and glucose tolerance were measured. Figure 1 shows dams on high-fat diets (HF45 and HF60) underwent the expected metabolic changes compared to dams fed the low-fat diet (LF10) in measures of body weights (Fig. 1A), body-fat percentage (Fig. 1B), and glucose tolerance test (Fig. 1C).

**Fig. 2:**
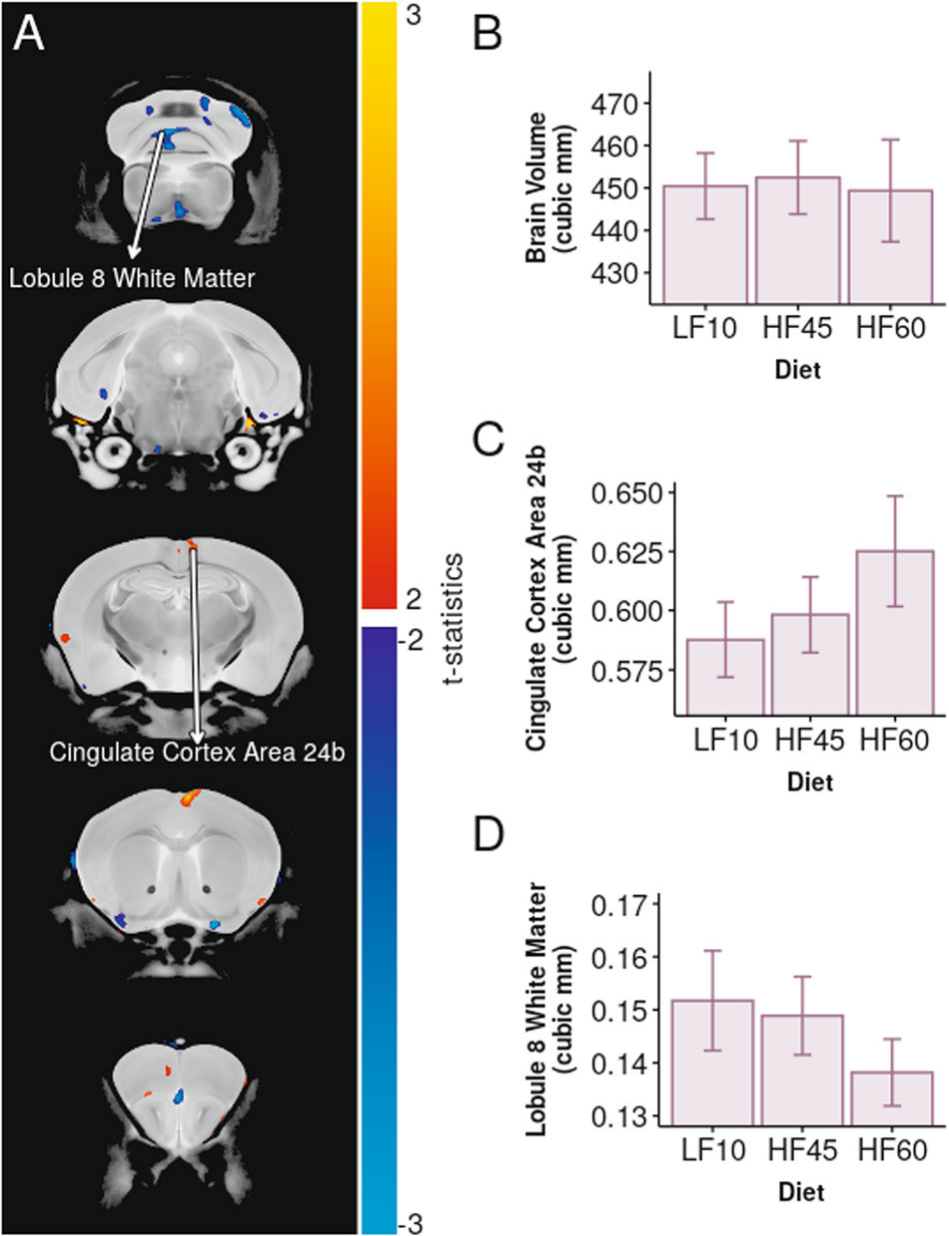
High-fat diet did not have a significant effect on the brain structure of dams. **A** Regional volume did not show significant negative or positive correlations with dietary fat-percentage (blue/turquoise and red/yellow colour scale, respectively). **B** Total brain volume was not significantly affected by diet (*F*_1,22_ = 0.003, *p* = 0.96). Cingulate cortex area 24b and White matter of cerebellar lobule 8 had the largest effects, but were not significant (ns). **C** Cingulate cortex area 24b showed increased volume with increased fat-percentage (*t*_22_ = 2.5, *p* = 0.02,FDR = 0.92, ns). **D** White matter of cerebellar lobule 8 showed decreased volume with increased fat-percentage (*t*_22_ = −2.13, *p* = 0.045,FDR = 0.98, ns).

After the 6-week acclimation period, all dams (LF10, HF45, HF60) continued on their respective diets throughout breeding, gestation and lactation (~12 weeks of dietary exposure). Subsequently, dams were killed for brain imaging. While a high-fat diet impacted dams’ physiology, it did not significantly affect their neuroanatomy – measured by brain volume (Fig. 2B), regional volume (Fig. 2A) and structural volume (examples Fig. 2C, D).

### Metabolic effects of maternal diet were ameliorated after high-fat diet was substituted with low-fat diet

**Fig. 3:**
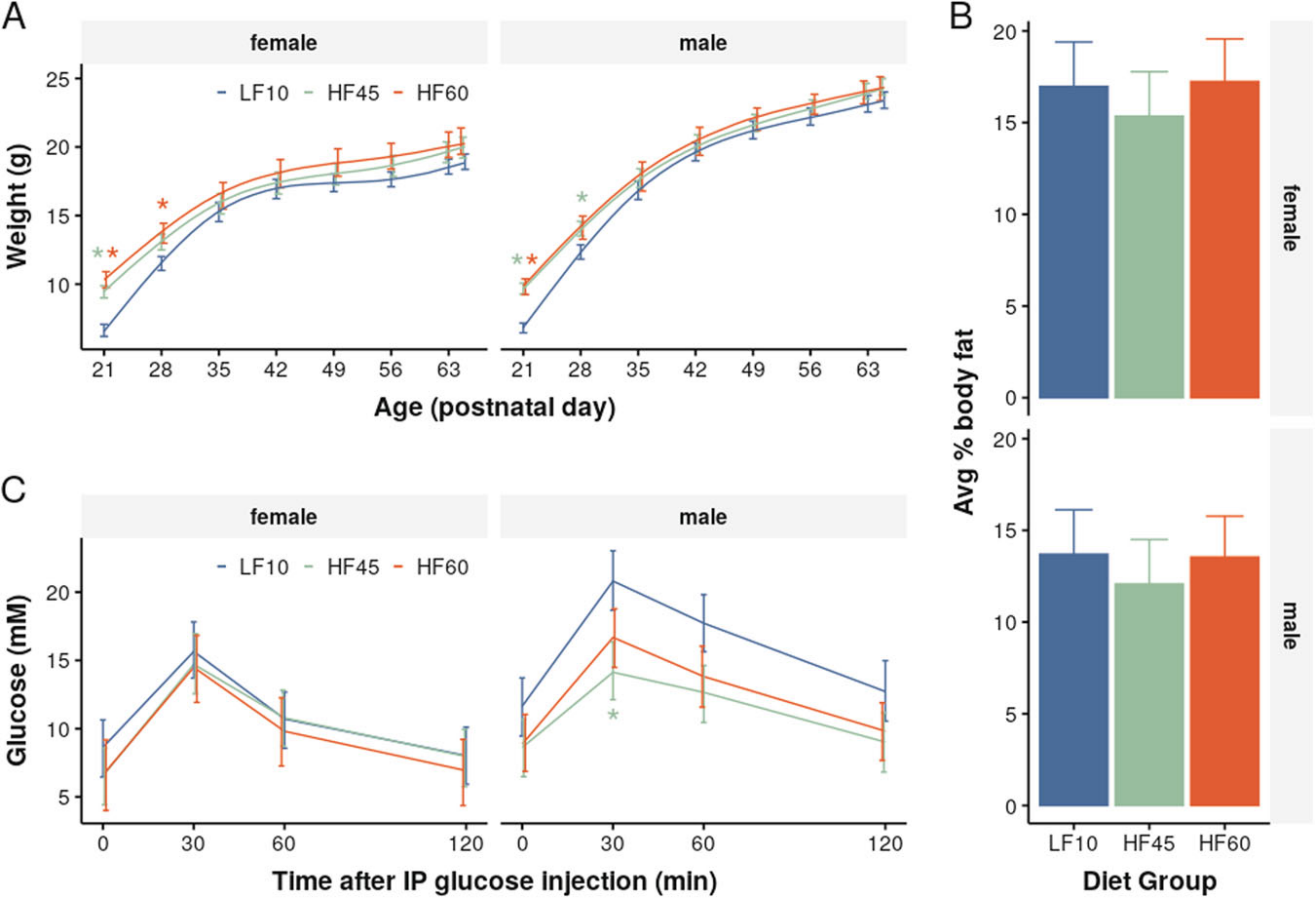
Offspring exposed to maternal high-fat diets showed amelioration of metabolic effects when weaned to a low-fat diet. **A** Body weights of HF45/HF60 mice normalised after being weaned onto the low-fat diet. There was a significant diet-time interaction (*χ*^2^_12_ = 71, *p* < 10^−9^) and confidence intervals of HF45/HF60 overlapped with LF10 mice after 2 weeks. **B** Body-fat composition, measured at P65, showed no significant effect of early-life diet (*F*_2,63_ = 1.20, *p* = 0.3). **C** GTT also showed no significant effect of early-life diet (*χ*_2,16_ = 23.6, *p* = 0.1). There was a decrease in blood glucose concentration 30 min after IP glucose administration in HF45 vs LF10 male offspring (difference = 6.63, *p* < 0.01). No significant decrease was found between the HF60 vs LF10 male offspring, and all female groups. All bars represent 95% confidence intervals. Asterisks represent a significant difference from LF10 (*p* < 0.05).

To limit experimental diet exposure to the in utero and perinatal period, offspring from all diet groups were placed on the low-fat diet at weaning on postnatal day 21 (P21). By early adulthood (P65), there was attenuation of all initial per group differences in body weight (Fig. 3A). Similarly, offspring exposed to a high-fat diet in early-life did not have significantly different body-fat composition at P65 compared to offspring raised solely on a low-fat diet (Fig. 3B). During GTT, there was no abnormal elevation of blood glucose concentration in HF45 and HF60 adult offspring (Fig. 3C), unlike their mothers (Fig. 1C).

### High-fat diet in early-life programmed brain structure changes in adulthood

**Fig. 4:**
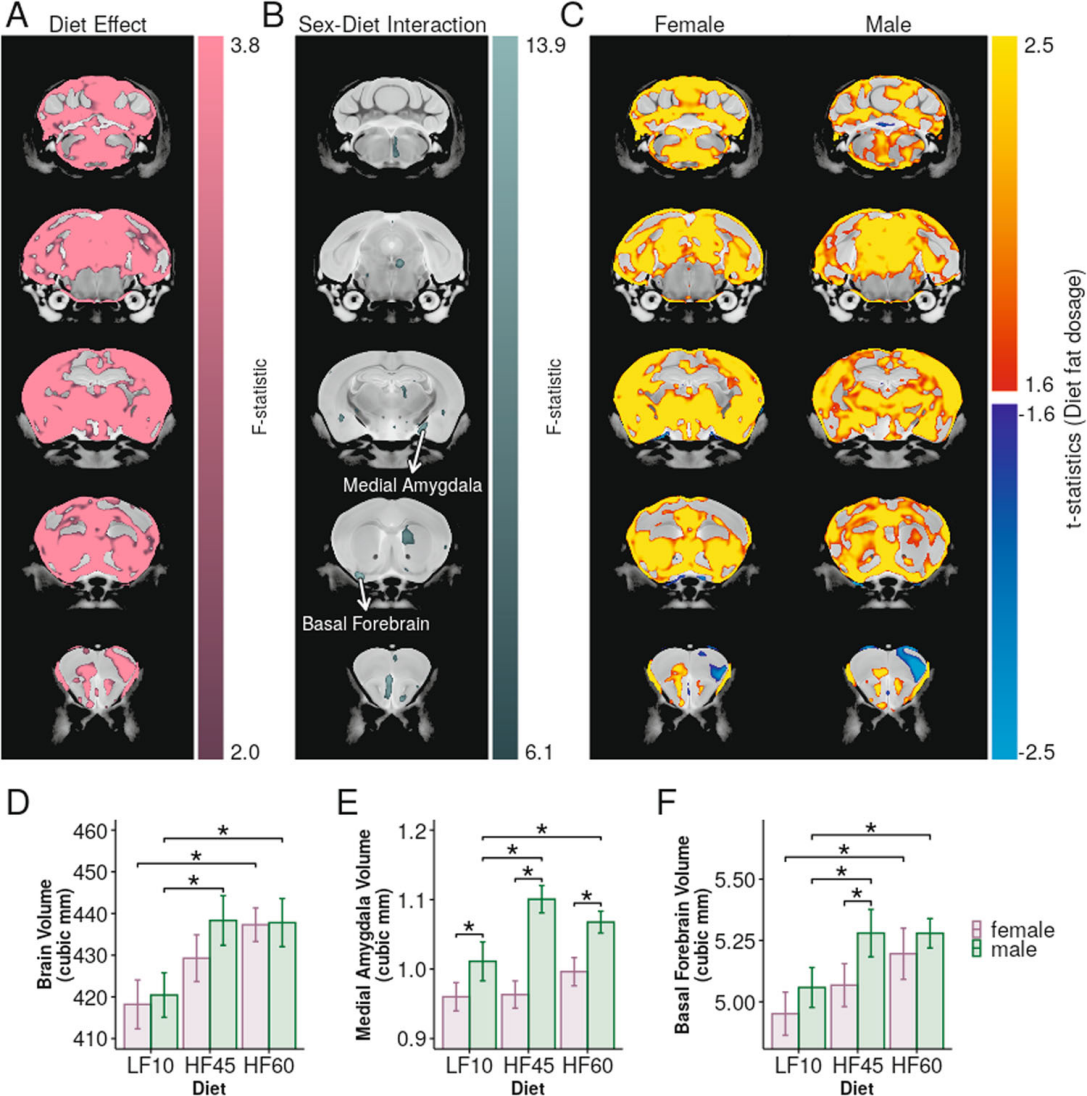
Offspring exposed to early-life high-fat diet exhibit extensive structural brain changes in adulthood. **A** Early-life high-fat diet was found to affect the volume of several regions throughout the brain. **B** In some brain regions, the effect of diet was modulated by sex. **C** Regional brain volume was largely correlated with early-life dietary fat-percentage (red/yellow colour scale), with some regions, such as the primary motor cortex, showing negative correlations (blue/turquoise colour scale). **D** Total brain volume was affected by early-life diet (*F*_2,95_ = 24, *p* < 10^−8^). Volumes of the medial amygdala (**E**) and the basal forebrain (**F**) were strongly impacted by diet (*F*_2,95_ = 13, *p* < 10^−4^, FDR < 10^−4^ and *F*_2,95_ = 14, *p* < 10^−5^, FDR < 10^−4^ respectively). The medial amygdala as a whole did not have significant sex-diet interactions (*F*_2,95_ = 8.9, *p* < 10^−3^, FDR = 0.051). Colour bars show 5% FDR at saturation and extend to 20% FDR to allow visualisation of cluster extent. Error bars represent 95% confidence intervals. Asterisks represent significant group comparisons (*p* < 0.05). Volume and effect-sizes for all brain structures are reported in Table S1.

Offspring were killed in early adulthood (P65) to image neuroanatomy. Their brains showed widespread volume changes in several regions due to high-fat diet exposure during gestation and lactation (Fig. 4 and Table S1), despite no such volume changes in dams (Fig. 2, effect-sizes compared in Fig. S1). The offspring brain structure changes were present despite transitioning them to a low-fat diet for 6 weeks post-weaning and amelioration of physiological abnormalities (Fig. 3). Early life high-fat diet exposure had a significant effect on both regional (Fig. 4A) and total brain volume (Fig. 4D). Since our study included 2 high-fat diets with differing fat percentage, we were able to explore possible dose-response effects. We found that dietary fat-percentage had a widespread positive correlation with the volume of several brain regions (Fig. 4C). The diet effect was found to be significantly different among males and females in a small number of isolated areas, such as voxels in the medial amygdala and the basal forebrain (Fig. 4B). However, these sex-interactions were only significant in subregions of these structures and not significant when analysing the bilateral structures as a whole (Fig. 4E, F).

### Brain regions sensitive to early-life high-fat diet were enriched with genes involved with behaviour and NDDs

**Fig. 5:**
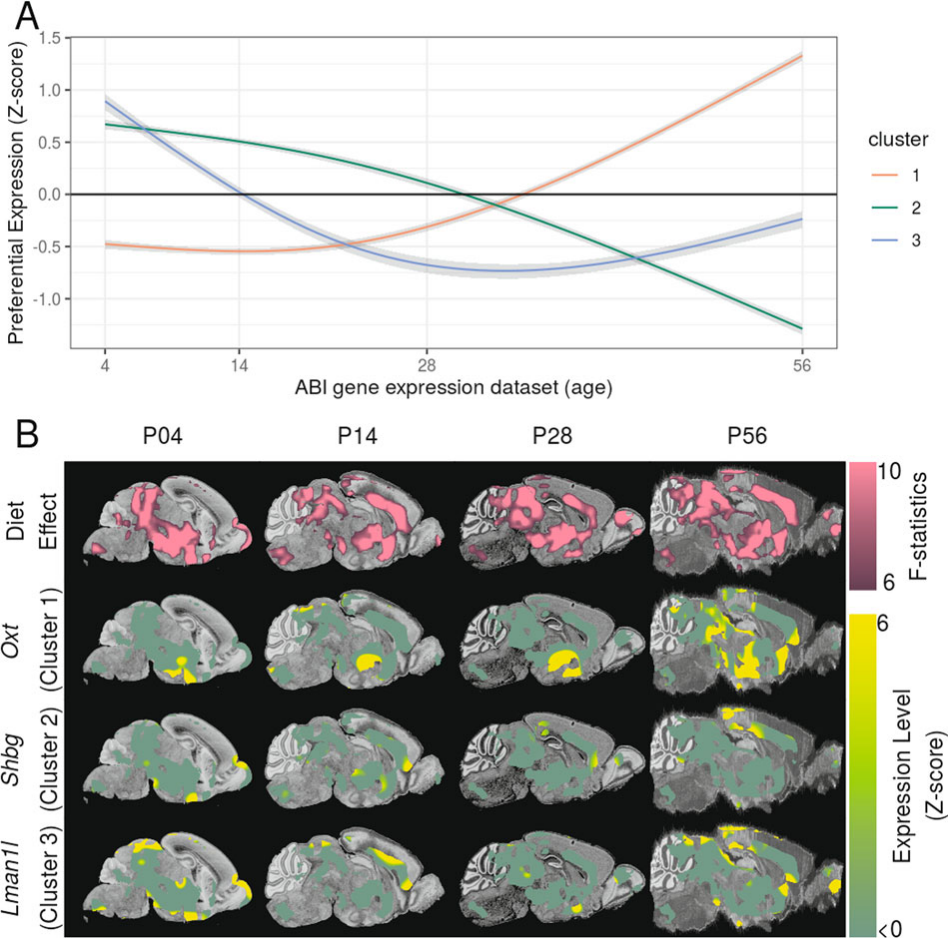
Genes expressed in brain regions sensitive to early-life diet over the course of mouse development. Brain regions where early-life diet had a highly significant effect (FDR < 0.01) on adult neuroanatomy define the ROI (**B** first row). The ROI was transformed to the nissl atlases defining the ABI gene expression dataset (background images in **B**) and the spatial expression of genes were quantified throughout the course of postnatal development. **A** Three clusters of spatio-temporal gene expression were found: genes with low expression in ROI during neonatal life and increased ~P28 (Cluster 1), and genes with high expression during neonatal life and decreased during ~P28 and ~P14 (Cluster 2 and 3, respectively). *Oxt* (Cluster 1) was chosen as a representative example of an ASD-associated gene (image row 2 in **B**). *Shbg* and *Lman1l* had the highest expression in Cluster 2 and 3 respectively and shown as representative examples (image row 3 and 4 in **B**). Shaded regions represent 95% confidence intervals. Gene expression fold-change maps were expressed as a *Z*-score and masked by the ROI for visualisation.

Maternal diet had a significant impact on the anatomy of several brain regions in the adult mouse (Fig. 5B, first row). We used the ABI Gene Expression atlas to identify genes preferentially expressed in this Region-of-Interest (ROI) throughout postnatal development^49^ (summarised in Table S2). Preferential expression was quantified using a fold-change measure – average gene expression in ROI divided by average gene expression in the whole brain. Upon clustering the fold-change values throughout development, we found three main clusters of genes: cluster 1 had low expression in ROI during neonatal life and increased during the juvenile period (P28), cluster 2 had high expression in ROI during neonatal life and decreased during the juvenile period (P28), and cluster 3 had high expression in ROI during neonatal life and decreased before weaning (P14; Fig. 5A). Sex-hormone-binding-globulin (*Shbg*) and Lectin-Mannose-Binding-1-Like (*Lman1l*) are genes with the highest expression in cluster 2 and 3, respectively, and were used as representative examples for illustration (Fig. 5B, third and fourth row).

Unlike the ABI development gene expression data, the ABI adult gene expression data spans the whole mouse genome, making it possible to run GO enrichment analysis and find biological processes enriched in preferentially expressed genes (Table S3). This is important as expression data from individual genes are known to have poor predictive relationship to neuroanatomy phenotype, however analysing multiple genes together (ex. GO enrichment) can reveal putative biological processes driving phenotypes^44^. Significant GO terms included synaptic signalling (GO:0099536, *q* = 0.03), feeding behaviour (GO:0007631, *q* = 0.02) and neuron differentiation (GO:0030182, *q* < 0.01).

Using the MGI database, we also examined human disease processes associated with homologous mouse genes. Genes associated with autism spectrum disorder (ASD) had significantly higher preferential expression in the ROI (defined as diet-sensitive brain regions) than the background set of all genes (*p*_adjusted_ = 0.017) and was the only significant disease term. The next most significant disease terms were schizophrenia and hydrocephalus (*p*_uncorrected_ = 0.01 and 0.02, *p*_adjusted_ = 0.15 and 0.28, respectively). To validate our finding, the SFARI database was queried to obtain a list of single-gene-mutant mouse models of autism. Similar to the analysis with the MGI dataset, genes associated with mouse models of autism had significantly higher preferential expression in brain regions sensitive to early-life diet (*p* < 10^−3^). For visualisation, *Oxt* (a member of cluster 1) was chosen as a representative of ASD-associated genes with increased spatial preferential expression (Fig. 5B second image row).

### Discussion

We demonstrated that high-fat exposure in early-life (based on maternal diet during gestation and lactation) caused structural changes in offspring brains that persisted into adulthood, even after the high-fat diet was removed and metabolic abnormalities attenuated. These data, coupled with the lack of diet effect on maternal brain structure, suggests that brain development may be particularly vulnerable to effects of high-fat diet during early-life. While there have been studies showing the effect of high-fat diet on older, juvenile mice, these studies generally focus on particular brain structures and behaviours^50,51^. To our knowledge, our study is the first to examine the whole brain and show that many structures in the developing brain are vulnerable to effects of maternal/early-life diet.

As proof of principle, we replicated existing findings that mice fed a diet consisting of 40–60% fat develop physical and metabolic abnormalities^13–15^, verifying that our diets and acclimation period had the desired effects on dams. After the 12-week course of high-fat diet, dams in our study (C57Bl6/J strain) did not have a neuroanatomical phenotype detectable by MR imaging. This is consistent with finding from Rollins et al using the C57Bl6/J x 129S1/SvImJ strain^52^.

Though this high-fat diet is used extensively in literature to explore the effects of high-fat intake, it is correspondingly low in carbohydrate content. Thus, separating the effects of low-carbohydrate intake from high-fat intake was not possible in this study. However, this aspect is likely not a substantial confound as varying carbohydrate intake with high-fat diets does not significantly impact level of obesity^53^. Furthermore, the control low-fat-diet (10% kcal from fat) used in both our study and extensively in literature^54–58^ is different from the standard mouse facility diet (18% kcal from fat). This may explain the overall low-brain volumes seen in low-fat-diet-fed offspring mice (Fig. 4D) compared to their mothers (Fig. 2B) and literature^59^. It may also explain differences in body-fat composition between offspring (Fig. 1B) and dams (Fig. 3B). While existing literature shows that phenotypic, metabolic and behavioural outcomes are similar across the 10%-fat and 18%-fat control diets^60^, our study demonstrates that the choice of control diet could affect neuroanatomy and should be taken into account.

Offspring exposed to maternal high-fat diet during the in utero and perinatal periods showed some recovery in metabolic measures after transitioning to the low-fat diet at weaning – similar to results in literature^61–63^. This metabolic recovery minimises the confounding effect of aberrant metabolism during adulthood on brain structure. Thus, the brain structure changes seen in the adult offspring were less likely due to ongoing metabolic abnormalities and, instead, more likely secondary to the longstanding effects of diet/metabolic milieu during in utero and perinatal life, which are important periods of organism development. Future studies could explore to what extent anatomical changes are directly due to metabolic effects or mediated by other factors like maternal care^64^. Furthermore, the brain regions identified in our study could be probed to examine the specific metabolites responsible for altered anatomy and explore possible targets for intervention. Finally, our study design can be readily extended to include in vivo MRI, which would provide additional information about when anatomical changes emerge and how long they persist^65^. These lines of investigations would provide valuable insight into the complex neural mechanisms responsible for the increased NDD risk in offspring exposed to maternal obesity.

The basal forebrain and medial amygdala are part of the extended-amygdala network and were found to be affected by early-life high-fat diet exposure. While behavioural assessment was outside the scope of this study, it is well-known that these structures have a role in reward-seeking behaviours and have been investigated extensively in the context of drug addiction^66^. More recently, studies have shown that the basal forebrain may also have an important role in food-seeking behaviour^67^. These structures are thought to play a role in aetiology of disorders such as ASD and anxiety^68^. The medial amygdala is also an important mediator of sex-specific behaviours^69^ and is known to be sexually dimorphic^33^. In some parts of the medial amygdala, we observed that the effect of diet on region volume was significantly modulated by sex. This preliminary finding should be investigated further given the sex differences in rates of NDDs.

ABI gene expression atlas^49^ was used to identify genes that are highly expressed in brain regions sensitive to early-life high-fat diet exposure. These candidate genes may drive the relationship between maternal diet and NDDs in offspring. The developing gene expression atlas revealed several genes that had high expression in these brain regions in neonatal life. Genes with the highest spatial preference in diet-sensitive brain regions in adulthood tended to be significantly involved with biological processes that impact organism feeding behaviour. Furthermore, genes associated with autism behaviours – in both mice and humans – were also found to have significantly higher spatial expression in brain regions impacted by early-life high-fat diet. These genes and related pathways may be important starting points for exploring genetic mechanisms linking maternal metabolic milieu to NDD risk in offspring.

In summary, exposure to high-fat diet in utero and during perinatal development had a significant impact on brain structure in adulthood. These effects were persistent despite a change to a low-fat diet that attenuated the metabolic effects of early-life high-fat diet exposure. The impacted brain regions included regions that play an important role in the neural circuitry of reward behaviours, and exhibited a high expression of genes associated with ASD. Our results demonstrate that the link between early-life high-fat diet exposure and risk for NDDs is also associated with brain structure changes, and provide strong evidence that the mouse can be used to investigate this relationship further.

## Supplementary information

Supplementary legends

Table S1

Figure S1

Table S2

Table S3

## Data Availability

All software used to perform the analysis is open-source. Code and data is available upon reasonable request.
