# Supplementary material for: Exposure to maternal high-fat diet induces extensive changes in the brain of adult offspring
Source: Transl Psychiatry. 2021 Mar 2;11:149. doi: 10.1038/s41398-021-01274-1 (PMC7925669; doi:10.1038/s41398-021-01274-1)
Supplement: Supplementary file 1 — Supplementary legends [file 41398_2021_1274_MOESM1_ESM.doc]

Supplementary information is available at TP’s website:

Table S1.xls: volume and effect-sizes for all brain structures

Table S2.xls: spatial gene expression preference in diet-sensitive brain regions

Table S3.xls: results of GO enrichment analysis on genes with high expression in diet-sensitive brain regions

Figure S1.doc: results of effect size correlations
