# Supplementary material for: Exposure to maternal high-fat diet induces extensive changes in the brain of adult offspring
Source: Transl Psychiatry. 2021 Mar 2;11:149. doi: 10.1038/s41398-021-01274-1 (PMC7925669; doi:10.1038/s41398-021-01274-1)
Supplement: Supplementary file 3 — Figure S1 [file 41398_2021_1274_MOESM3_ESM.doc]

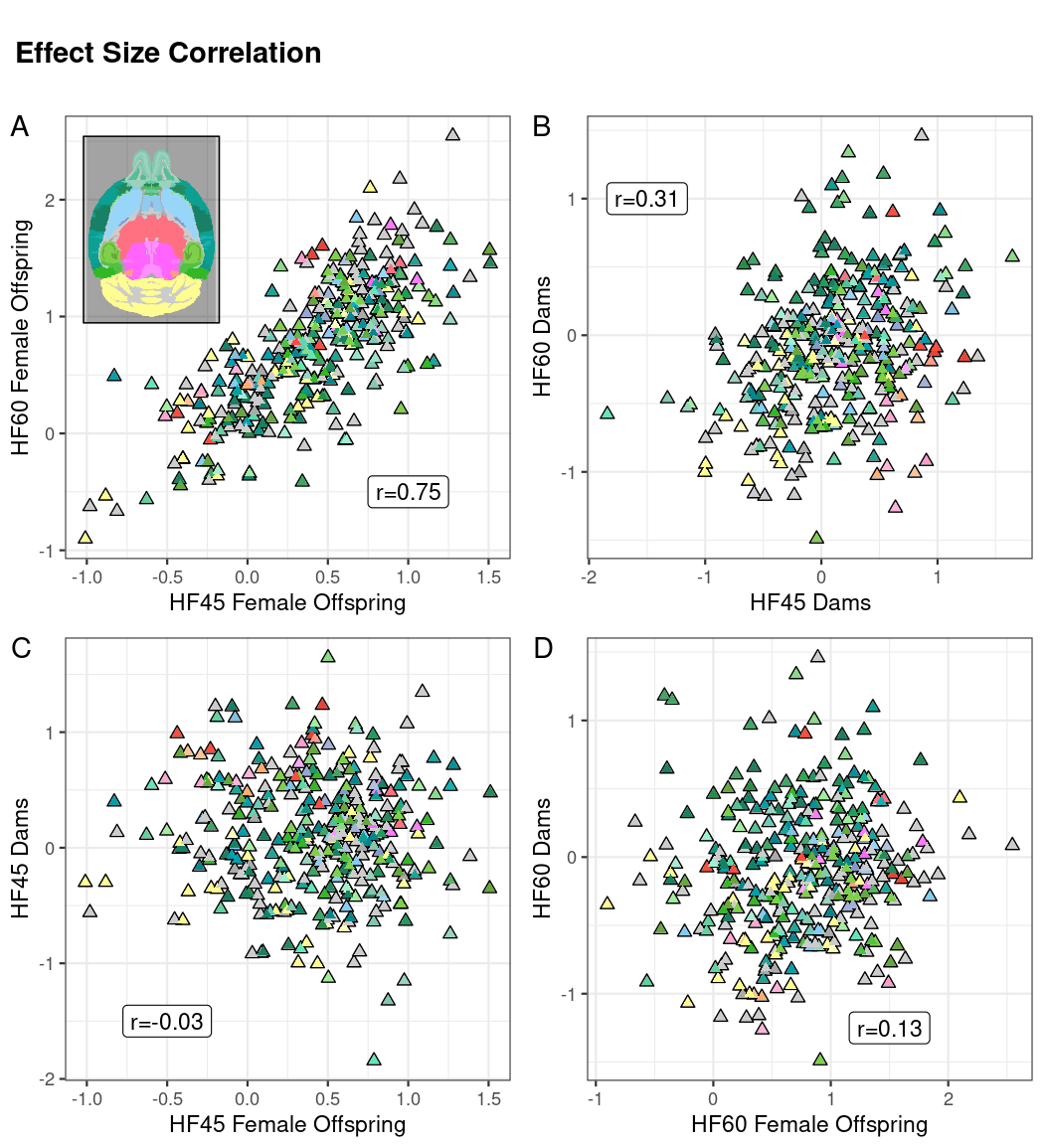


**Supplementary Figure 1:** Correlation in effect sizes computed for all brain structures in female offspring and dams, and for all diets. Effect sizes (Cohen’s *d* with pooled standard deviation) were computed for each of the 336 brain structures comparing the high-fat diets (HF45 and HF60) versus low-fat diets (LF10). The effect sizes were also computed for female offspring and dams separately. In order to compare effect sizes in dams and offspring, male offspring were excluded to remove confounding variable of sex (i.e. only female offspring were used in the computation). The correlation across brain structures between experimental groups were plotted and Pearson correlation (*r*) was provided as an inset. (A) Effect sizes across brain structures (represented with triangle points) were correlated between HF45 female offspring (x-axis) and HF60 female offspring (y-axis), indicating similar brain regions in offspring were affected by both high-fat diets. Points colour represents the structure colour convention used by Allen Brain Institute’s Mouse Atlas (horizontal slice provided as inset). (B) Effect sizes across brain structures were also correlated between HF45 dams and HF60 dams. Poor correlations were seen when comparing effect sizes across brain structures of female offspring (x-axis) and dams (y-axis), for both (C) HF45 and (D) HF60 diets. This indicates that different brain regions were affected by high-fat diet in offspring and dams.
